# Comment on: "Serologic screening for viral infections among blood donors: a study in a blood bank in southern Brazil"

**DOI:** 10.1590/1806-9282.20250246

**Published:** 2025-07-07

**Authors:** Fatma Yilmaz

**Affiliations:** 1 Ankara Etlik City Hospital, Department of Hematology – Ankara, Turkey.

Dear Editor,

I read with interest the article "Serological screening for viral infections among blood donors: a study in a blood bank in southern Brazil" published in the 70th issue of *Revista da Associação Médica Brasileira*. I wanted to add my comment on the subject of this article and the practices in Turkey.

Blood and blood component transfusions are a supportive treatment that reduces mortality and morbidity when performed in the correct indication and at the correct time. Blood and blood components are products obtained from voluntary donations, the only source of which is human^1^.

During the blood and blood component donation process, information identification, registration and query form filling, physician evaluation, rejection criteria query, immunohematological and microbiological evaluations, and detailed examinations are performed. Despite all these detailed and meticulous evaluations during this process, the risk of infection with blood and blood component transfusion is still one of the most important complications^2^.

Although there are differences between countries around the world, the microbiological agents screened during the blood donation process are hepatitis B virus (HBV), hepatitis C virus (HCV), human immunodeficiency virus (HIV), *Treponema pallidum* (syphilis), *Trypanosoma Cruzi* (Chagas disease), human T-lymphotropic virus (HTLV ½), plasmodium (malaria), cytomegalovirus (CMV), and West Nile virüs^3^.

When we look at Turkey, routine HBV (1983), HCV (1996), HIV (1987), and syphilis screening were done. Malaria screening was done between 1992 and 1997^2^.

Belanda et al. reported that serological changes and donor fidelity rates of viral infections screened in blood donations were compared in two consecutive 5-year periods (2013–2017 and 2018–2022). HBV (anti-hepatitis B core antibody [HBc], hepatitis B surface antigen [HbsAg]), HCV (anti-HCV), HIV (anti-HIV 2, HIV antigen test p24), HTLV (anti-HTLV ½) serological markers were used. A decrease was reported in all virus serologies screened, with the highest rate in HIV serology and the lowest rate in anti-HBC serology. When looking at fidelity data, a high rate of 70% was observed. A significant decrease in all screened viral serologies and a high donor loyalty rate are indicators of the blood center's success^4^.

In the study, both HbsAg and anti-HBc were examined for HBV screening. In order not to miss active HBV patients in the window period, it is recommended to check anti-HBc immunoglobulin G (IgG) in the HbsAg-negative period^5^.

However, despite all these serological screenings, there is still a possibility of donors in the seronegative period. In this period with false-negative seroprevalence, the use and spread of nucleic acid amplification (NAT) tests have led to a further decrease in the risk of blood transfusion infection transmission.

NATs are tests that detect the presence of viral nucleic acid (DNA/RNA). Low-level viral nucleic acids formed in the sample become detectable by amplifying the target nucleic acid region. It has been used in the routine screening of volunteers who donate from Red Crescent centers in Turkey since 2014^6^. NAT will be used more widely in future studies.

The tests used in screening are a dynamic process with increasing sensitivity, with new ones being added to developing technologies. Each country should revise and develop screening programs before utilizing blood and blood components by conducting regular analyses and considering newly emerging infectious agents.

## Data Availability

The datasets generated and/or analyzed during the current study are available from the corresponding author upon reasonable request.
